# Regulatory implementation of Optical Coherence Tomography as an analytical technique in pharmaceutical fillings

**DOI:** 10.3389/fmed.2025.1693159

**Published:** 2026-01-07

**Authors:** Jesús Alberto Afonso Urich, Raymar Andreina Lara Garcia, Matthias Wolfgang, Johannes Khinast

**Affiliations:** 1 Institute of Process and Particle Engineering, Graz University of Technology, Graz, Austria; 2 Research Center Pharmaceutical Engineering GmbH, Graz, Austria

**Keywords:** GMP compliance, Optical Coherence Tomography (OCT), pharmaceutical filling, regulatory modernization, technologies implementation

## Abstract

Optical Coherence Tomography (OCT) has emerged as a powerful non-destructive imaging tool capable of delivering high-resolution cross-sectional images of drug formulations, particularly useful for evaluating coating uniformity, detecting defects and characterizing multi-layered structures in solid dosage forms. Despite OCT potential, its widespread acceptance has been limited due to the lack of pharmacopeial monographs and specific regulatory guidelines. In recent years, growing regulatory interest in advanced analytics has prompted increased attention to method validation, alignment with Quality by Design (QbD) principles and compliance with Good Manufacturing Practices (GMP). This work provides an integrated perspective on the development, implementation and regulatory evaluation of OCT in the pharmaceutical industry. It reviews the implementation process and the evolving regulatory framework surrounding OCT in pharmaceutical applications, along with practical considerations for its adoption. As interest in OCT on the part of regulatory bodies grows, the pharmaceutical industry is moving toward broader engagement, emphasizing the need for standardization and eventual inclusion of OCT methodologies in regulatory frameworks. With continued collaboration between the industry stakeholders, regulatory agencies and standard-setting organizations, OCT is positioned to become an integral component of modern pharmaceutical quality control strategies.

## 1 Introduction

The pharmaceutical industry is continuously evolving, integrating novel analytical technologies to improve quality control, reduce time-to-market and enhance process understanding. Regulatory agencies, including the U.S. Food and Drug Administration (FDA) (1) and the European Medicines Agency (EMA) (2), encourage innovation in analytical methodologies while ensuring compliance with current Good Manufacturing Practices (cGMP) (3, 4). Many health agencies have initiated and actively promoted the adaptation process of Process Analytical Technology (PAT), redefining pharmaceutical manufacturing and quality assurance for the future (5–7).

Quality by Design (QbD) is today one of the main concepts in pharmaceutical development. It applies a systematic approach based on predefined objectives, thorough process and product understanding, risk assessment and effective process control (8, 9). Grounded in sound science and Quality Risk Management (QRM), this approach aims to enhance the robustness and predictability of pharmaceutical manufacturing (10). The integration of new analytical techniques plays a crucial role in achieving these objectives, ensuring better characterization and monitoring of pharmaceutical products. To align with regulatory expectations, such technologies must also demonstrate suitability for inclusion within control strategies and lifecycle management frameworks (11).

In this context, Optical Coherence Tomography (OCT) for analysis of coatings and films has emerged as a valuable non-destructive imaging tool in pharmaceutical quality control. OCT enables high-resolution cross-sectional imaging, which is particularly useful for analyzing the coating uniformity, detecting defects in solid dosage forms and characterizing multi-layered drug formulations (12–19). Together with other in-line PAT methods, such as Infrared (IR) and Raman spectroscopy (20–26), OCT has the potential to enhance product quality and manufacturing control strategies. Importantly, the regulatory relevance of OCT arises from its capability to provide real-time, non-destructive data that support enhanced process understanding and evidence-based decision-making (5, 27, 28).

Despite these advancements, adopting new analytical technologies in the pharmaceutical industry creates regulatory challenges, including validation requirements, standardization and integration into existing quality frameworks (29–31). The aim of this work is to provide a regulatory perspective on the implementation of innovative analytical technologies, with a particular focus on OCT. The discussion below addresses the OCT application in pharmaceutical testing, the related validation strategies and potential regulatory pathways for broader acceptance.

## 2 Applications of OCT in pharmaceutical development

Optical Coherence Tomography has unique imaging capabilities that enable detailed structural and morphological analysis of pharmaceutical products, making it a versatile tool at various stages of drug development and manufacturing. Its high-resolution, cross-sectional imaging functionality provides valuable insights into the internal structure of dosage forms that cannot be easily achieved through conventional methods. From a regulatory standpoint, these capabilities directly support enhanced process understanding as outlined in ICH Q8–Q10 and align with expectations for advanced analytical tools within GMP/PAT frameworks (5, 10, 11, 28, 30).

A primary and well-documented application of OCT in pharmaceutical development is a non-destructive analysis of solid oral dosage forms, particularly tablets and capsules. OCT is capable of providing cross-sectional images with a micrometer-resolution that allow for real-time assessment of Critical Quality Attributes (CQAs), such as the coating thickness, integrity and uniformity (13, 17–19, 32). This is especially relevant for coated dosage forms, where regulatory agencies emphasize the need for reliable characterization to ensure the intended functional performance, particularly for delayed- or enteric-release systems (33, 34).

Several scientific studies have demonstrated that OCT can accurately quantify the coating thickness and its variability (12–14, 18, 32, 35, 36). However, OCT also enables the detection and quantification of sub-visible defects, such as the layer deformations and delamination (37), the presence of voids, inclusions and layer constrictions (38), the surface roughness on complex surfaces (39), and in-situ monitoring of membrane swellability/film porosity (40), which are often missed when conventional analytical methods are applied. Compared to classical destructive techniques (e.g., cross-sectional microscopy, dissolution profiling), OCT offers the advantage of maintaining the sample integrity, enabling repeated measurements in the same unit and supporting statistical analysis across large sample sets.

Beyond endpoint testing, OCT has also been investigated for its potential in PAT applications, particularly during spray-coating processes. For example, real-time OCT monitoring of film coating dynamics enables the visualization of coating growth trends and early detection of process deviations (13, 16, 41). Such implementations support the industry’s movement toward continuous manufacturing and real-time release testing (RTRT), although the latter is still under regulatory consideration and research. In this context, OCT contributes to the type of real-time, science-based control strategies encouraged by regulatory agencies as part of modernization initiatives (27, 28).

Furthermore, OCT has been proven capable of differentiating between individual coating layers, making it suitable for the analysis of complex multilayered oral solid dosage forms (14). This is particularly valuable during the formulation development and scale-up, when understanding the layer integrity and homogeneity is critical for ensuring batch-to-batch reproducibility and therapeutic performance. Clear visualization of multilayer structures may also support regulatory expectations for demonstrating product understanding during lifecycle management, especially on CMC sections of the dossier (42).

Additionally, OCT has been applied in other areas of pharmaceutical development beyond oral dosage forms. For example, it has been used to measure the dimensions of vaginal inserts in hormone replacement therapy (37, 38), as well as to assess drug-loaded polymers in intravaginal rings, in which the thickness of the polymer layer governs the drug release profile (43). These diverse applications underline OCT’s potential as a flexible analytical tool capable of supporting regulatory submissions across a variety of dosage forms.

While the OCT benefits are clear, several challenges to its full-scale integration into routine manufacturing and regulatory submissions remain. These include the need for standardized calibration procedures, validated image analysis algorithms and defined acceptance criteria for the selected metrics. Nonetheless, the growing body of literature supports the inclusion of OCT as a robust analytical tool for the characterization and quality assurance of a broad range of pharmaceutical dosage forms. Addressing these gaps will be essential for achieving broader regulatory acceptance and facilitating future integration of OCT into formal guidance and compendial standards.

## 3 Regulatory considerations for OCT implementation

### 3.1 Compliance with data integrity and GMP requirements

The use of OCT in regulated environments requires adherence to stringent data integrity standards. Imaging data, particularly when collected in real time, must be captured, stored and managed in compliance with ALCOA + principles that ensure that all data are Attributable, Legible, Contemporaneous, Original and Accurate (44). OCT systems must also meet the requirements of 21 CFR Part 11 or equivalent EU Annex 11 (44) regulations for electronic records and signatures. GMP compliance also extends to equipment qualification and software validation. OCT instruments intended for GMP use must undergo installation qualification (IQ), operational qualification (OQ) and performance qualification (PQ), with all associated documentation maintained according to regulatory expectations (45). Software platforms used for image acquisition and analysis must demonstrate validated functionality and audit trails (3, 4, 46). A particular challenge with regard to OCT is the management of large volumes of imaging data, which may complicate version control, traceability and archiving. Risk-based approaches to data retention, audit readiness and periodic review must be established to ensure long-term compliance. Clear data governance strategies and risk-based approaches to retention and audit readiness are therefore essential for long-term compliance.

### 3.2 Analytical method validation

Analytical method validation is a critical process of ensuring that an analytical procedure is fit for its intended purpose in the context of pharmaceutical development and regulatory compliance (47). The methodology employed must be rigorously defined and derived via comprehensive, scientifically justified method development and optimization studies (48). Validation not only confirms the reliability and consistency of the procedure but also establishes predefined acceptance criteria for system suitability tests, which are essential for verifying the method performance prior to a routine analysis.

The validation process must be executed in strict accordance with a predefined validation protocol (47). The outcome of the validation studies should be thoroughly documented in a formal validation report and integrated into regulatory submissions under Section 3.2.P.5.3 of the Common Technical Document (CTD) (42), in alignment with ICH and regional regulatory guidelines.

Since OCT relies on interferometric imaging principles, traditional validation parameters outlined in ICH Q2(R2) (47) (e.g., accuracy, precision, specificity, linearity, range and robustness) may require adaptation. The challenge lies in the inherently different nature of imaging data compared to spectroscopic or chromatographic data. Unlike conventional analytical methods, OCT produces spatial and morphological information, which requires the development of specific performance metrics, such as the resolution fidelity, contrast differentiation, depth penetration and repeatability of structural measurements.

Furthermore, the qualitative and semi-quantitative aspects of OCT imaging demand rigorous calibration strategies and possibly the development of surrogate quantitative indicators (e.g., thickness measurements as proxies for dose uniformity). Currently, there is no universally accepted validation framework for OCT in pharmaceutical applications, which is a significant barrier to regulatory harmonization. As a result, companies exploring OCT are often compelled to design tailored validation studies, which may or may not align with the regulatory expectations. Standardization of terminology, performance criteria and reporting formats will be essential to ensure consistent interpretation and reproducibility.

### 3.3 Regulatory pathways for acceptance

Pathways for regulatory acceptance of OCT-based methodologies are currently fragmented and highly depend on the individual agency’s engagement. Early scientific advice meetings and pre-submission discussions with agencies, such as the FDA (49, 50) and the EMA (2, 51), are vital to clarifying the expectations and reducing the risk of rejection or requests for Supplementary Data.

A key strategic consideration is to initially introduce OCT as a supportive tool rather than as a primary method of specification testing in development dossiers. This allows regulators to become familiar with the technique while providing the applicants (sponsors) with an opportunity to demonstrate reliability, relevance and added value. Over time, as evidence of robustness and regulatory utility accumulates, OCT may be transitioned into primary testing roles.

It is also advisable to reference well-documented case studies and peer-reviewed publications within regulatory filings to establish precedent and scientific justification. Where possible, alignment with ICH guidelines, especially Q8, Q9 and Q10 (10, 11, 30) and QbD principles can further strengthen the regulatory argument for the use of OCT. This structured approach helps frame OCT within existing regulatory expectations and facilitates predictable agency interactions.

### 3.4 Integration into existing regulatory frameworks

Integrating OCT into established regulatory frameworks remains a complex endeavor. Unlike IR and Raman spectroscopies that benefit from inclusion in pharmacopeial standards (52–54) or a regulatory guideline (55, 56), OCT lacks a formal compendial chapter. This absence of standardized methods and acceptance criteria limits the comparability of results across laboratories and hinders their broader regulatory acceptance.

In addition, OCT’s classification within the regulatory lexicon is still evolving. It is not yet clearly defined whether OCT should be considered a PAT tool (5, 16, 17), a quality control release test or a characterization method. The lack of categorization complicates its placement in the CTD for drug submissions and may lead to variability in regulatory agency responses.

To facilitate the OCT integration, a multi-stakeholder approach is required, involving regulatory bodies, pharmacopeias, technology developers and industry. Harmonized guidance documents that outline acceptable performance characteristics, use cases and reporting formats for OCT are critical for enabling consistency in regulatory submissions. Such harmonization would also support future compendial inclusion and global regulatory alignment.

### 3.5 Roadmap for implementation of regulatory guideline

As discussed above, regulatory agencies have established specific channels for engaging into a dialogue on emerging scientific policies that align with the adoption of novel technologies. In this context, both the FDA and the EMA have launched strategic initiatives to facilitate the integration of innovative approaches into pharmaceutical development and manufacturing.

The FDA’s Emerging Technology Program (ETP) (1) offers a proactive mechanism, which allows applicants to engage early with the agency regarding the development and implementation of novel manufacturing technologies, including advanced analytical techniques such as OCT. Through the ETP, pharmaceutical companies can propose using OCT in specific applications, e.g., real-time monitoring of coating thickness and structural characterization of complex dosage forms. Subsequently, they can receive scientific feedback concerning the regulatory expectations, validation strategies and potential pathways of incorporation into regulatory submissions. The collaborative and non-binding nature of the ETP fosters innovation by enabling scientific dialogue and risk-based decision-making outside the constraints of formal application processes.

According to the FDA (49), the ETP requires the submission of a brief proposal (approximately five pages) that includes the following elements:

1. A short description of the proposed emerging technology
2. An explanation why the technology is substantially novel and warrants inclusion in the ETP
3. An explanation of how the technology may improve the product safety, identity, strength, quality or purity
4. A summary of the development plan, including potential regulatory or technical challenges
5. A projected timeline for submission of a regulatory application (e.g., IND, ANDA, NDA, BLA or DMF)

In principle, it is expected that the Marketing Authorization Holder (MAH) will be responsible for submitting the application, either independently or in collaboration with the technology owner or, in the OCT case, the provider of the OCT platform used. The MAH is accountable for filing regulatory submissions, including the IND, ANDA, NDA and BLA. While the capability of OCT as an analytical technology is well-established in the scientific literature, its regulatory application must be articulated by describing the analytical procedure and its relevance to the pharmaceutical product in question.

Similarly, the EMA’s Quality Innovation Group (QIG) (2) serves as a platform to support the evaluation and regulatory acceptance of novel technologies within the EU framework. The QIG provides early scientific advice and creates a structured pathway for companies to present innovative quality concepts, including OCT-based approaches. By fostering a dialogue between regulators, industry and academic experts, the QIG aims to ensure consistent and efficient assessment of innovation across the EU, improving regulatory predictability.

It is important to note that the QIG’s focus may vary annually based on the strategic priorities of the European Commission. For example, in 2025, the group prioritized personalized medicine. Although OCT may not directly align with the QIG’s current focus on personalized medicine, it can still be relevant in certain contexts. Proposals that highlight its application in individualized therapies, such as precision-controlled drug release or patient-specific dosage forms, may be considered within the QIG’s scope (2).

Both the ETP and the QIG exemplify regulatory openness to new technologies, provided their implementation is scientifically justified and supported by robust data. In this context, OCT presents a compelling opportunity: its non-destructive high-resolution imaging capabilities are consistent with the overarching goals of improving the product quality, manufacturing efficiency and patient-centric innovation.

In addition to the FDA’s ETP and EMA’s QIG, regulatory authorities in Japan and China have also established mechanisms to support the integration of innovative pharmaceutical technologies. In Japan, the Pharmaceuticals and Medical Devices Agency (PMDA) facilitates early dialogue on novel technologies through its scientific consultation services and the Science Board framework (57–60). While not dedicated solely to manufacturing innovation (57, 58), these pathways could allow for discussion of advanced analytical methods such as OCT, particularly when aligned with QbD or continuous manufacturing strategies. Similarly, in China, the Center for Drug Evaluation (CDE) under the National Medical Products Administration (NMPA) (61) offers pre-communication mechanisms that enable sponsors to discuss emerging technologies within the context of product development (62). Although China has not formalized a specific “emerging technology” program, recent regulatory reforms and growing alignment with ICH guidelines demonstrate increasing openness to pharmaceutical innovation (63). In both countries, early engagement with regulatory agencies through existing scientific advice frameworks remains critical to facilitate the acceptance of novel technologies such as OCT.

The development plan should be guided by the MAH’s implementation strategy and include a clear justification for the use of OCT, supported by documented evidence and a comprehensive gap analysis. To fully leverage regulatory programs, applicants must also define the intended use of OCT, its role within the control strategy, and its alignment with the QbD and lifecycle management principles (11, 64–66). This justification should be supported by method validation data, comparability studies and performance metrics that demonstrate the reliability and reproducibility of the technique.

One key gap identified in the regulatory landscape is the lack of a formal guideline for OCT analytical procedures. To address this, we have developed a draft guidance document (see Supplementary materials) titled “*Development and Submission of Optical Coherence Tomography Analytical Procedures*.” It is based on the structure and logic of existing guidance documents for other emerging technologies (56) and is intended to facilitate more of a structured and productive engagement with regulatory authorities.

Looking ahead, formal recognition of OCT in ICH guidelines or its inclusion in pharmacopeial standards would significantly streamline regulatory acceptance and facilitate global harmonization (67). As members of the ICH (68), regulatory authorities in the United States, the European Union, Japan, and China are increasingly aligned in their support for science and risk-based approaches to pharmaceutical quality. Until such recognition is achieved, proactive engagement with the FDA and EMA through the ETP and QIG, respectively, remains a strategic pathway (1, 2). Similarly, early dialogue with Japan’s PMDA and China’s NMPA through their consultation frameworks (57, 58) offers a viable route to advancing the regulatory integration of OCT in pharmaceutical development and manufacturing on a global scale.

## 4 Conclusions and future outlook

Optical Coherence Tomography is a transformative advancement in pharmaceutical analytical science, offering real-time high-resolution non-destructive imaging that complements and extends existing quality assessment methodologies. Its capacity to visualize internal microstructures without altering the sample makes it particularly well-suited for evaluating the coating uniformity, detecting defects and characterizing complex or layered dosage forms.

Despite being in the early stages of regulatory recognition, it has been demonstrated that OCT can be widely applicable across pharmaceutical development, from early formulation design to in-line process monitoring. OCT’s alignment with contemporary quality paradigms, including QbD, PAT and continuous manufacturing, underscores its relevance to evolving regulatory expectations and supports the shift toward science- and risk-based decision making.

However, a broader regulatory adoption of OCT will require a concerted effort to establish validated, reproducible methodologies and harmonized performance criteria. The absence of standardized guidance, variability in instrumentation and limited integration into compendial frameworks remain critical barriers to its widespread implementation. Addressing these challenges will necessitate collaborative engagement of industry stakeholders, regulatory authorities and standard-setting organizations.

To support the regulatory acceptance, future efforts should prioritize the development of robust validation protocols, the generation of multi-center performance data, and the incorporation of OCT methodologies into regulatory submissions and pharmacopeial standards. Emerging areas, such as advanced image analytics, artificial intelligence integration (69) and applications beyond solid dosage forms, are promising avenues for extending the OCT applicability. These innovations may further strengthen OCT’s regulatory value by improving measurement consistency, standardization and interpretability.

In summary, OCT has significant potential to enhance the pharmaceutical product quality and regulatory compliance. Realizing this potential will depend on sustained research, cross-sector collaboration and regulatory innovation aimed at integrating OCT into the scientific and operational fabric of pharmaceutical development and manufacturing. As regulatory frameworks evolve, OCT is well-positioned to become a key analytical tool supporting modernized, data-rich quality assessment.
