# Supplementary material for: Regulatory implementation of Optical Coherence Tomography as an analytical technique in pharmaceutical fillings
Source: Front Med (Lausanne). 2026 Jan 7;12:1693159. doi: 10.3389/fmed.2025.1693159 (PMC12819765; doi:10.3389/fmed.2025.1693159)
Supplement: Supplementary file 1 [file Supplementary_file_1.docx]

Supplementary Material

# Development and Submission of Optical Coherence Tomography Analytical Procedures Guidance for Industry (Draft)

## INTRODUCTION

This guidance is intended to provide recommendations to aid the development, validation and use of analytical procedures for the evaluation of performance test dissolution of drug products based on Optical Coherence Tomography (OCT). The recommendations apply to new drug applications (NDAs), abbreviated new drug applications (ANDAs), supplemental NDAs and ANDAs for small-molecule drugs. They will help Marketing Authorization Holders (MAHs) compile the information and data necessary to support the analytical methodologies. Those analytical methodologies should be aligned with the International Conference on Harmonisation (ICH) Guideline Q2(R2) on Validation of Analytical Procedures, for developing and validating analytical methods (1).

Furthermore, since OCT is a technique to be applied as a process analytical technology (PAT) tool, this guidance contains recommendations to the MAHs for applying the concepts described in the FDA Guidance for Industry PAT - A Framework for Innovative Pharmaceutical Development, manufacturing, and Quality Assurance (2). Additionally, the use of mathematical models for OCT analytical methodologies should follow the principles stated in regulated monographs, e.g., USP 〈1039〉 Chemometrics (3). The OCT implementation at commercial manufacturing sites must be in accordance with the current good manufacturing practice (cGMP) regulations CFR - Code of Federal Regulations Title 21 parts 210 and 211 (4) for drug products, for example. This guidance pertains only to the development and validation of OCT analytical procedures and does not provide recommendations concerning the setup, qualification, maintenance or calibration of OCT instruments.

If the MAH chooses a different approach than those recommended in this guidance, we encourage them to discuss the matter with the appropriate *Regulatory Agency* quality assessment staff before submitting the application.

This document is intended to clarify existing regulatory expectations and should not be construed as establishing legally enforceable responsibilities. It is not legally binding unless explicitly incorporated into a binding contract or regulation. The *Regulatory Agency* guidance documents and this document should be viewed only as recommendations, unless specific regulatory or statutory requirements are cited. The use of the word *should* in the *Regulatory Agency* guidance means that something is suggested or recommended, but not required.

## BACKGROUND

Concerning OCT as an analytical technique, each NDA and ANDA applicant must include the required analytical procedures to determinate direct variables, such as the coating thickness and homogeneity, or indirect variables (via mathematical models), such as the dissolution rate of the drug product (for functional coatings) and the porosity (5). Data must be available to establish that the analytical procedures used in testing meet proper standards of accuracy, sensitivity, specificity and reproducibility and are suitable for their intended purpose (6).

Data for analytical procedures verification or validation should be provided in the corresponding sections of the application in the ICH M2 eCTD: Electronic Common Technical Document Specification.

## ANALYTICAL METHOD DEVELOPMENT

At the start of OCT methodology development, method parameters, such as specificity, linearity, range, accuracy, limit of detection (LOD), limit of quantification (LOQ) and precision should be assessed. A systematic approach is recommended to examine the method's robustness. Additionally, an initial risk assessment should be in place for the evaluation of OCT methodology and the formulation’s compatibility (e.g., presence of titanium dioxide). Typically, when these aspects are considered, the chance of understanding the parameters' effects on the eventual method performance is higher.

## GENERAL MODES OF TESTING FOR OCT

For OCT testing, the following modes of measurement are commonly used for process understanding, monitoring and control:

- off-line with the sample analyzed away from the process stream (e.g., the coating thickness test of the final dosage form in a quality control lab)
- at-line with the sample removed and analyzed close to the process stream (e.g., measuring the coating thickness of a tablet via an OCT analyzer located next to the pan coater).
- in-line with the sample not removed from the process stream (e.g., an in-line OCT monitoring system installed in a pan coater or fluid-bed coater taking continuous images of the ongoing coating process).

## CONTENT OF ANALYTICAL PROCEDURES

The analytical procedures should be described in sufficient detail (including all special aspects) to be reproducible for any competent analyst. Aligned with the Guidance for industry Analytical Procedures and Methods Validation for Drugs and Biologics (7). Generally, the essential sections to be included are:

### Principle/Scope

A description of the OCT principle of measurement on a particular sample type (e.g., enteric-coated tablet) is required.

### Apparatus/Equipment

The equipment to be used, as well as all components, should be qualified and within an internal qualification program.

### Operating Parameters

Defined, validated and qualified settings of the OCT equipment (e.g., the distance between the probe and the sample, the relative scanning velocity, the acquisition rate, the field of view, the angle of image acquisition, etc.) should be described.

### Sample Presentation

If applicable, the sample presentation should be described in detail to minimize variability in the interpretation and ensure an unequivocal analysis. Examples of sample presentation setups are rotating disc systems, flow-cell assemblies for continuous analysis and static configurations paired with a scanning beam. Clear specification of the sample orientation, positioning stability and interface conditions (e.g., optical contact or refractive index matching) is essential to ensure consistent image acquisition and data comparability across laboratories.

### Standard Control Materials

Detailed description of current standardized reference material(s) (e.g., films) to be used should be provided. A certification should be available that includes important factors (e.g., the refractive index).

### Procedure

A step-by-step description of the method (e.g., the equilibration times, the number of oversampling, the scanning time, the standard measurement, the blanks, the samples, etc.). If the OCT method is used for in-line process measurements, a clearly defined system suitability procedure must also be included to ensure consistent performance under real-time conditions.

### System Suitability

Confirmatory test(s) procedures and parameters to ensure that the system (the equipment, the attached sensors and the sample presentation) will function correctly as an integrated system at the time of use should be described. The system suitability acceptance criteria have to be defined in the analytical procedure and justified accordingly.

### Calculations

Representative examples of calculations for data analysis, including mathematical transformations and scientific justification for any correction factors to be applied, should be provided.

### Data Reporting

The instrumental capabilities and acceptance, which are consistent with the results report, must be presented. The results (e.g., the thickness, the homogeneity of coating, % of dissolution) should be indicated in a specific format following the use of significant digits according to the American Society for Testing and Materials (ASTM) E29 (8).

The OCT procedure should be included in section 3.2.P.5.2 of the CTD.

## REFERENCE MATERIALS

In the context of the OCT analytical technique, a reference material is defined as a product characterized by a metrologically valid procedure for one or more specified properties (e.g., thickness), accompanied by a certificate indicating values ​​of the specified property, its associated uncertainty and a definition of its metrological traceability.

The MAH should provide information about any reference materials intended to be used in the application, including qualification test reports and certificates of analysis. A description of the reference material, source, storage conditions, directions for safe use and documented shelf life must be provided. The data presented in the certificate of analysis must be derived from a calibration exercise and processed using a robust statistical program to ensure accuracy and reliability. The methodology for its determination must be specific, and the certificates must show the confidence interval, the uncertainty, the predictive value, the expanded uncertainty, the mean value and the deviation from the mean.

The reference materials (e.g., films of defined properties) used for method validation must be tested and certified by a GxP-compliant laboratory. If a general polymer is used as a reference, a justified risk analysis to evaluate the impact on the measurement and an assessment of the differences between the reference and sample material are recommended.

## ANALYTICAL METHOD VALIDATION

Analytical method validation is the process of demonstrating that an analytical procedure is suitable for its intended purpose. The methodology should be established and obtained via a scientifically based method development and optimization studies (7). Validation is also applied to establish acceptance criteria for the system proficiency tests that are used to verify the analytical procedure prior to the analysis. It must be carried out following the validation protocol, which states the analytical performance characteristics to be verified for the various types of analytical procedures. The results must be documented in the validation report and section 3.2.P.5.3 of the CTD.

### Validation Characteristics

The following validation characteristics are applicable for OCT testing:

- **Specificity.** This parameter indicates the degree to which other substances present in the matrix of the formulation interfere with the determination of the attribute via OCT, considering that the technique is limited for identification purposes. The refractive index of the material must be certified unequivocally since the determination principle is highly dependent on this parameter (9). The reference material films should be tested both in the absence and in the presence of the product formulation. The resulting measurements should then be compared to assess whether the formulation components interfere with the OCT signal.
- **Linearity and range.** To evaluate linearity, the analytical results obtained via OCT should be compared to its reference materials within the specified range, depending on the final product characteristics. The linearity is acceptable if the correlation coefficient is close to 1 and the y-intercept is close to 0. The appropriate range depends on the measured attribute.
- **Accuracy.** The accuracy of the OCT procedure is evaluated by comparing the results with the reference materials. Depending on the product characteristics, for a one-point specification, a determination at 70%, 100% and 130% levels is sufficient. A cross-validation approach using a separate analytical technique, such as (electron-) microscopy, is recommended.
- **Precision (repeatability and intermediate repeatability).** Samples within the expected range should be evaluated multiple times to establish the standard deviation. Intermediate repeatability should be assessed by varying analysts and testing days using the same sample set. The resulting variability (e.g., standard deviation or relative standard deviation) should be comparable to that observed under repeatability conditions, indicating consistent performance across different conditions. Furthermore, the method should evaluate changes that might occur during the coating process within the operational range of the equipment.
- **Limit of Detection and Limit of Quantification.** If the OCT-based analytical procedure is applied near its detection or quantification limit, both the LOD and the LOQ must be determined separately. Each should be established using its respective reference standard set.
- **Robustness.** Given the critical impact of process variations on the coating process trajectory, it is essential to include an assessment of these variations in the validation of the analytical procedure. This ensures that potential changes within the equipment's operational range are systematically evaluated and documented.

### Validation Acceptance Criteria

The validation acceptance criteria should be justified using risk assessment tools (e.g., FMEA, decision tree, risk matrix, etc.). A scientific evaluation should consider the criticality of the measurement for the safety and efficacy of the patient (10). Furthermore, the specific acceptance criteria for each validation parameter should be consistent with the intended use of the method (CQA) (11).

For questions regarding suitable validation approaches for analytical procedures or submission of information not addressed in this guidance, the MAH should consult with the appropriate *Regulatory Agency* quality assessment staff.

## DEVELOPMENT OF OPTICAL COHERENCE TOMOGRAPHY MODELS

A fundamental component of OCT measurement is the generation of structural models based on pixel-based image analysis. This involves identifying and counting pixels that correspond to specific material features, such as the boundaries of a coating layer, in cross-sectional OCT images. The intensity and location of each pixel are analyzed to distinguish between coating material and background or core structures, often based on contrast thresholds or segmentation algorithms.

Mathematical models are then applied to translate these direct OCT measurements (e.g., coating thickness, layer integrity, or porosity) into indirect responses related to CQAs, such as dissolution or drug release profiles. These models are typically constructed using regression algorithms or other mathematical expressions and require robust calibration and validation using reference datasets.

### Development of a Calibration Set

The development of a calibration set is an important aspect of constructing an OCT model. The calibration samples furnish the information for the calibration set. The calibration set should contain samples that: (1) include appropriate working ranges for the parameters to be analyzed; (2) allow to investigate possible sources of variability (e.g., a variation in the processes, the analyzer, the physical properties of the materials); and (3) encompass anticipated variations in the process parameters (e.g., in-design-space parameters) that can influence the response.

Calibration samples should resemble the samples that are indicative of the commercial process as closely as possible. Calibration samples from batches that are either generated at the intended commercial scale or are indicative of commercial process are ideal since they reflect the anticipated process uncertainty. Additional samples can be created in laboratory-scale settings if the samples acquired using the commercial-scale method do not provide sufficient variability.

The MAHs should consider the following, while creating calibration samples:

- Differences between the samples and the actual product (e.g., the coating surface roughness, the inter-coating space, the presence of pigments in the coating formulation, differences in the refractive index and the absorbance properties) may alter the calibration findings.
- The material properties of the samples should assure a response that is comparable to that of the actual product on the commercial scale. If required, a statistical approach is recommended for assessing the equivalency. The image acquisition should sufficiently reduce the encountered differences. Furthermore, an evaluation of several approved lots and suppliers is recommended.
- Environmental uncertainty can be addressed by monitoring the image acquisition under different conditions or setting the environmental conditions for the measurement. Additionally, the sample presentation should be evaluated since imaging could be affected.
- It is critical to standardize the calibration set since it allows further extension to other instruments or testing locations in the future.

### Presentation of Samples

Regardless of the type of attributes measured via the OCT technology, the sample presentation to the instrument, the scanning velocity, the distance to the probe and the angle could influence the resulting tomograms and, therefore, the response. The MAHs should select and set the most appropriate configuration for the measurement. Several considerations should be made in terms of justifying the selection:

- The selected configuration should reflect the calibration and commercial production sample images.
- Replicate acquisition is recommended as a reproducibility verification of the selected positioning.
- The influence of image acquisition parameters, such as the scanning angle and velocity, should be evaluated and clearly defined to ensure reproducibility.
- The OCT system should consistently operate within the same wavelength range, including the fixed center wavelength and the bandwidth.
- The field of view (FOV) must be specified and kept constant during the data acquisition. Consistent vertical positioning of the sample in the tomogram is also critical for achieving comparable and interpretable results.

These parameters should be included in the method documentation and verified during the method qualification and transfer.

### Mathematical Model

In any OCT analytical procedure, the models are regularly built using the pixels from a settled image. An image of an established size (e.g., 1024x1024 pixels) is produced, describing cross-sections of the dosage form.

The MAHs should address the following topics while developing the mathematical models:

- Evaluation of the OCT image should confirm that the dosage form is properly positioned and fully visible within the field of view. This includes verifying pixel intensity profiles and selecting appropriate measurement parameters using the analysis software. Proper positioning of the sample in the tomogram is critical for ensuring the accurate extraction of relevant structural features. To enhance reliability, feature extraction should be focused on regions with sufficiently high pixel intensities, while establishing a threshold to exclude areas below the defined minimum intensity. This approach supports consistent and reproducible analysis of the targeted structural attributes.
- An algorithm is selected to provide a best fit to the mathematical expression (e.g., an ellipse on concave tablets) of the assumed surface shape. Sometimes this could be pre-set by the software.
- Additional expressions or parameters may be considered when conducting the thickness measurements. In this context, such attributes as the minimum and maximum values, can be used to define the length and thickness profiles more precisely, enabling a more detailed characterization of the layer variability.
- Since the algorithm selection highly depends on the user configuration parameters, their combination with the implemented acquisition method defines the algorithm. The user interface parameters of the model calibration, which are intrinsically dependent on each pharmaceutical product or the mode of testing, must be established.
- Reference samples, such as polymer material films with controlled and well-characterized variation in thickness or surface properties, should be used when developing or applying a mathematical model. These samples help establish the model’s ability to detect and quantify meaningful differences. Verification of a 90% confidence interval for the measurement is recommended.

## VALIDATION OF OPTICAL COHERENCE TOMOGRAPHY MODELS

The most common approach to creating a quantitative calibration model of OCT is via an internal validation set, with the model providing a set of results that are compared to the validation set. The residuals are considered in the calculation of the standard error of prediction (SEP).

One criterion for model optimization is the root mean square error of SEP. The best model for the analytical technique generally has a reasonable (and justifiable) error, which reduces the error's sensitivity to slight changes in the sample characteristics or the model parameters.

The standard error of calibration (SEC) after the optimization process should guarantee that the measurement is performed with the required (low) uncertainty for the intended application of analytical procedure.

## LIFE CYCLE MANAGEMENT

At the start of the development of OCT analytical procedures an enhanced approach from the basic framework of analytical lifecycle management should be used (12). The continuous evaluation of the risk to quality must be based on scientific knowledge and, ultimately, linked to the patient safety (10).

In addition, a risk-based approach to revalidation of existing analytical methods may have to be considered when the manufacturing process changes during the product’s life
cycle (7). Any changes to OCT procedures must be handled according to cGMP and documented under the manufacturing facility quality system (13).

Regarding the post-approval changes, the *Regulatory Agency* proposes several mechanisms of how to proceed depending on the potential impact of the method and the final outcome.

### Major Changes

Major changes imply a potentially high impact on the OCT method performance, as in the drug product. Those should be reported via a Prior Approval Supplement (PAS)

Some examples are:

- Changes in the coating material, where the refractive index has to be altered and the methodology has to be re-validated.
- Implementation of OCT analytical procedures for real-time-release testing (RTRT) of the drug product, as well as significant variations of an already implemented procedure.
- Replacement of an OCT analyzer that has not proven any equivalence via cross-validation.
- Application of new calibration models.

### Moderate Changes

Moderate changes are those that normally have a high impact on the OCT analytical procedure but a low impact on the drug product quality or a medium-to-low impact on the procedure and a significant-to-high impact on the drug product quality. Those should be addressed in the CBE-30 Supplement.

Some examples include:

- Applying a new mathematical model for the transformation of response from OCT.
- Changing the supplier of coating material, when the refractive index could be affected and eventually the methodology should be re-validated or at least verified.
- Implementing broader specifications within an already validated range of the analytical procedure.

### Minor Changes

Minor changes are those that have a medium impact on the OCT method performance and a low impact on the drug product. Those should be reported via Annual Report (AR), and the minimal ones should be reported in Pharmaceutical Quality System (PQS).

Some examples include:

- Changing the pretreatment of the acquired spectral data that are equivalent to the originally approved procedure.
- Implementing tighter specifications within an already set validation range of the analytical procedure.

## ABREVIATIONS

**ANDA.** Abbreviated New Drug Application

**AR.** Annual Report

**ASTM.** American Society for Testing and Materials

**CBE-30.** 30 Days supplement

**CFR.** Code of Federal Regulations

**eCTD.** Electronic Common Technical Document

**FMEA.** Failure Mode Effect Analysis

**GMP.** Good Manufacturing Practices

**ICH.**International Council for Harmonisation

**LOD.** Limit of Detection

**LOQ.** Limit of Quantification

**MAH.** Marketing Authorization Holder

**NDA.** New Drug Application

**OCT.** Optical Coherence Tomography

**PAS.** Prior Approval Supplement

**PQS.** Pharmaceutical Quality System

**RTRT.** Real Time Release Testing

## REFERENCES

1. International Council for Harmonisation. Guideline Q2 (R2) Validation of Analytical Procedures [Internet]. 2022 [cited 2024 Oct 25]. Available from: https://www.ema.europa.eu/en/documents/scientific-guideline/ich-guideline-q2r2-validation-analytical-procedures-step-2b_en.pdf

2. FDA Center for Drug Evaluation and Research. Guidance for Industry PAT - A Framework for Innovative Pharmaceutical Development, manufacturing, and Quality Assurance. 2004.

3. USP43-NF38. 〈1039〉 Chemometrics [Internet]. [cited 2022 Jan 5]. Available from: https://doi.usp.org/USPNF/USPNF_M2345_02_01.html

4. U.S. Food and Drug Administration, Center for Drug Evaluation and Research C for BE and R. CFR - Code of Federal Regulations Title 21 parts 210 and 211. [Internet]. [cited 2024 Nov 15]. Available from: https://www.accessdata.fda.gov/scripts/cdrh/cfdocs/cfcfr/cfrsearch.cfm

5. U.S. Food and Drug Administration, Center for Drug Evaluation and Research C for BE and R. CFR - Code of Federal Regulations Title 21 Part 314.50 and 314.94 [Internet]. [cited 2024 Jan 5]. Available from: https://www.accessdata.fda.gov/scripts/cdrh/cfdocs/cfcfr/cfrsearch.cfm?fr=314.50

6. U.S. Food and Drug Administration, Center for Drug Evaluation and Research C for BE and R. CFR - Code of Federal Regulations Title 21 Part 165(e) and 194(a) [Internet]. [cited 2024 Jan 5]. Available from: https://www.accessdata.fda.gov/scripts/cdrh/cfdocs/cfcfr/CFRSearch.cfm?CFRPart=211

7. FDA Center for Drug Evaluation and Research. Analytical Procedures and Methods Validation for Drugs and Biologics Guidance for Industry [Internet]. 2015 [cited 2024 Sep 23]. Available from: https://www.fda.gov/media/87801/download

8. American Society for Testing and Materials. E29 - Practice for Using Significant Digits in Test Data to Determine Conformance with Specifications [Internet]. West Conshohocken, PA: ASTM International; 2022 Apr. Available from: http://www.astm.org/cgi-bin/resolver.cgi?E29-22

9. Wolfgang M, Peter A, Wahl P, Markl D, Zeitler JA, Khinast JG. At-line validation of optical coherence tomography as in-line/at-line coating thickness measurement method. Int J Pharm. 2019 Dec 15;572:118766.

10. International Council of Harmonization. Guideline Q9 Quality Risk Management [Internet]. 2014 [cited 2020 Nov 22]. Available from: https://www.ema.europa.eu/en/documents/scientific-guideline/international-conference-harmonisation-technical-requirements-registration-pharmaceuticals-human-use-ich-guideline-q9-quality-risk-management-step-5-first-version_en.pdf

11. USP43-NF38. 〈1225〉 Validation of Compendial Procedures [Internet]. 2021 [cited 2024 Feb 22]. Available from: https://doi.usp.org/USPNF/USPNF_M99945_04_01.html

12. USP-NF 〈1220〉 Analytical Procedure Life Cycle [Internet]. [cited 2024 Oct 18]. Available from: https://doi.org/10.31003/USPNF_M10975_02_01

13. International Council of Harmonization. ICH guideline Q10 on pharmaceutical quality system [Internet]. [cited 2020 Nov 22]. Available from: https://www.ema.europa.eu/en/documents/scientific-guideline/international-conference-harmonisation-technical-requirements-registration-pharmaceuticals-human_en.pdf
